# Certificate-of-need laws and substance use treatment

**DOI:** 10.1186/s13011-022-00469-z

**Published:** 2022-05-18

**Authors:** James Bailey, Thanh Lu, Patrick Vogt

**Affiliations:** 1 Providence College Department of Economics, 1 Cunningham Sq, Providence, RI 02918 USA; 2 Department of Population Health Sciences at Weill Cornell Medical College, 1300 York Avenue, New York, NY 10065 USA

**Keywords:** Certificate-of-need laws, Substance use treatment, Healthcare payments, National Survey of substance Abuse treatment services

## Abstract

**Background:**

Certificate-of-need (CON) laws in place in most US states require healthcare providers to prove to a state board that their proposed services are necessary in order to be allowed to open or expand. While CON laws most commonly target hospital and nursing home beds, many states require CONs for other types of healthcare providers and services. As of 2020, 23 states retain CON laws specifically for substance use treatment, requiring providers to prove their “economic necessity” before opening or expanding. In contrast to the extensive academic literature on how hospital and nursing home CON laws affect costs and access, substance use CON laws are essentially unstudied.

**Methods:**

Using 2002–19 data on substance use treatment facilities from the Substance Abuse and Mental Health Services Administration’s National Survey of Substance Abuse Treatment Services, we measure the effect of CON laws on access to substance use treatment. Using fixed-effects analysis of states enacting and repealing substance use CON laws, we measure how CON laws affect the number of substance use treament facilities and beds per capita in a state.

**Results:**

We find that CON laws have no statistically significant effect on the number of facilities, beds, or clients and no significant effect on the acceptance of Medicare. However, they reduce the acceptance of private insurance by a statistically significant 6.0%.

**Conclusions:**

Policy makers may wish to reconsider whether substance use CON laws are promoting their goals.

**Supplementary Information:**

The online version contains supplementary material available at 10.1186/s13011-022-00469-z.

## Introduction

Substance use disorders (SUDs) are chronic health conditions and characterized by clinically significant impairment, including health problems, disability, engaging in unintended risky behaviors, and failure to meet major responsibilities at work, school, or home, related to the use of alcohol and/or illicit drugs [1]. These conditions impose substantial costs on both affected individuals and the nation as a whole in terms of lost lives, lost health, lost productivity, and crime. Data from the National Survey on Drug Use and Health suggest that, in 2019, 7.4% of the population (20.4 million people aged 12 or older) had a SUD in the past year, and even this may be an underestimate [2]. In the same year, there were 70,630 deaths from drug overdose [3]. Although SUDs are preventable and treatable health conditions and treatment has been shown to reduce SUDs and their associated harms, a treatment gap continues to exist [4]. Common SUDs are alcohol, cannabis, stimulants, and opioids [5]. Estimates suggest that less than 20% of those with SUDs received any treatment in the past year [6]. Furthermore, these conditions are most prevalent among low-income and uninsured individuals [7], implying that taxpayers finance a large share of the costs associated with SUDs. For example, while Medicaid covered 16% of the nonelderly adult population, Medicaid covered 38% of nonelderly adults with SUD in 2017 [7].

Certificate-of-need (CON) laws in place in most US states require healthcare providers to prove to a state board that their proposed services are necessary in order to be allowed to open or expand. Conover and Bailey [8] provide extensive background on their history, intent, and effects. While CON laws most commonly target hospital and nursing home beds, data from the American Health Planning Association show that some states require CONs for up to 28 separate types of healthcare providers and services. In the face of a national opioid epidemic, 22 states and the District of Columbia retain CON laws specifically for SUD treatment, requiring providers to prove their “economic necessity” before opening or expanding. In contrast to the extensive academic literature on how hospital and nursing home CON laws affect costs and access, substance use CON laws are essentially unstudied. Only one prior article has studied the effect of substance use CON laws, and only one outcome has been studied. Noh and Brown [9] found that CON laws led to fewer SUD treatment facilities per capita.

Given that SUDs place a great burden on both the affected individual and society with the annual economic costs of SUDs being $555 billion in 2019 dollars [4], understanding how CON laws for SUD services affect access to treatment is important. One of the commonly cited barriers to accessing services is the lack of available treatment providers or programs [6]. If CON laws promote access to care for poor and underserved communities, as one of its intended justifications, CON laws may increase access to treatment among low-income populations. However, if CON laws act as restrictions on entry into a market and reduce competition, CON laws may decrease access to treatment through reduced facilities and/or available beds.

Using data on CONs from the American Health Planning Association and the Mercatus Center together with 2002–19 data on treatment facilities from the Substance Abuse and Mental Health Services Administration’s (SAMHSA’s) National Survey of Substance Abuse Treatment Services (N-SSATS), we measure the effect of CON laws on access to substance use treatment. Using fixed-effects analysis of states enacting and repealing substance use CON laws, we measure how CON laws affect the number of SUD treatment facilities, beds per capita, and clients per capita in a state. In addition, we measure the effect of CON laws on the forms of payment that treatment facilities accept, with a large share of cash-only facilities serving as a proxy for excess demand.

### Data

Data on CON laws come from the American Health Planning Association (AHPA) [10] and the Mercatus Center [11]. Different states wrote their CON laws to apply to different types of treatments, capital equipment, and health facilities. From 1992 to 2016, AHPA tracked which states required a CON for each of 28 different types of healthcare, ranging from acute-care hospital beds, MRIs, neonatal intensive care units, to SUD treatment facilities. The most common types of CON restrictions in 2016 were for acute-care hospital beds (27 states), long-term acute-care beds (26 states), and ambulatory surgery centers (26 states). The data sources make it clear that CON typically applies to both proposed new facilities and to expansions of existing facilities, particularly adding new beds. The sources do not make it clear which states require CON for all substance use treatment and which states exempt outpatient facilities. While AHPA has not updated its data since 2016, other organizations began tracking CONs more recently. The National Council of State Legislatures [12] and the Institute for Justice [13] provided snapshots of 2019, and the Mercatus Center provided the most recent CON census for 2020 [11].

**Fig. 1:**
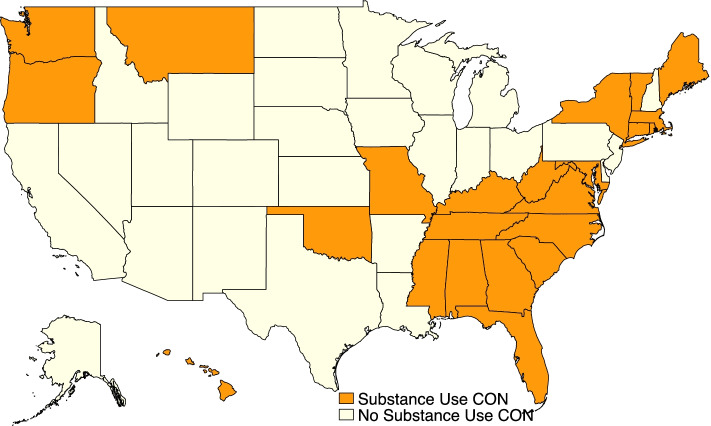
States with Substance Use–Treatment CON Laws in 2020. Note: Created by authors using data from Mitchell, Philpot, and McBirney [11]

When there were discrepancies between data sets, we examined the relevant state statutes and regulations to determine when exactly states passed or repealed substance use CON laws; see Additional file 1: Appendix Table 1 for details. During the period of our study, our data show 2 states repealing substance use CON laws (Alaska and Nevada), 1 state adding one (Kentucky), and 2 states both adding and repealing them (Connecticut and Washington DC). Figure 1 shows which states had substance use CON laws in place as of 2020.

All data on SUD treatment facilities are from the 2002–19 National Survey of Substance Abuse Treatment Services (N-SSATS), a survey conducted by SAMHSA [14]. The survey is completed by specialty SUD treatment facilities, with response rates of approximately 89%. A specialty SUD treatment facility is defined by SAMHSA as a hospital (including VA), residential facility, outpatient treatment facility, or other facility with an SUD treatment program that offers the following services: outpatient, inpatient, or residential rehabilitation treatment; detoxification; opioid-use treatment; and halfway-house services. Treatment in specialty settings accounts for approximately 70% of SUD expenditures in 2015 [14].

The survey asks a wide variety of questions including how many clients the facility saw last year, how many beds it has, and what forms of payment it accepts. However, not every question was asked every year. While the survey began in 1997, we use data from 2002 and on, as such omissions were particularly common between 1997 and 2001. While facility-level responses are publicly available, we use the state-level aggregate data provided by N-SSATS, given that our goal is to determine the effects of CON laws at the state level. The N-SSATS provides raw counts of the total number of facilities, beds, clients, and facilities accepting various forms of payment in each state; we have rescaled these variables. We calculate the percentage of facilities accepting certain forms of payment (Medicaid, Medicare, private insurance) by dividing the number of facilities that accept each form of payment by the total number of facilities in a state. We calculate per capita versions of the facilities, beds, and clients variables using data on total state population from the Current Population Survey: facilities per 100,000 residents, beds per 100,000 residents, and clients per 1000 residents. For the facilities-per-state variable, we use the number of facilities eligible to be surveyed, regardless of whether they responded to the survey. Client counts in the N-SSATS represent a snapshot of the number of clients on an average day, not annual totals.

State-level demographic control variables come from the Current Population Survey and were collected via the Integrated Public Use Microdata Survey [15]. We also control for two relevant state-level policy variables. Data on Prescription Drug Monitoring Programs (PDMP) come from Horwitz et al. 2021 [16]. Data on health insurance benefit mandates for drug treatment are from the Blue Cross Blue Shield Association [17]. These laws require private health insurers to cover drug treatment; see Bailey 2022 for a discussion of how they affect healthcare finance [18]. Table 1 shows the summary statistics for all variables used in our analysis.

**Table 1:** Summary statistics 2002–19

Variable	Obs	Mean	Std Dev	Min	Max
Facilities per 100 k	918	5.63	2.58	1.56	17.07
Beds per 100 k	763	36.72	17.66	7.46	136.60
Clients per 1 k	813	4.48	2.70	1.04	53.35
% Accept Private Ins	918	0.71	0.14	0.29	0.98
% Accept Medicare	918	0.37	0.13	0.09	0.73
% Accept Medicaid	918	0.62	0.18	0.15	0.95
Substance Use CON	918	0.49	0.50	0.00	1.00
PDMP	918	0.13	0.34	0	1
SUD Mandate	918	0.76	0.43	0	1
Population (mil)	867	5.95	6.71	0.49	39.31
Median Age	867	37.33	2.40	27.50	44.90
Income (thousands)	867	41.44	9.52	23.21	81.88
% White	867	80.20	13.83	17.77	97.80
% Black	867	11.29	11.15	0.09	63.27
% Hispanic	867	10.33	9.88	0.10	52.72
% Asian	867	4.35	7.72	0.07	70.78
% Male	867	49.08	0.83	46.60	52.09
% In Poverty	867	12.79	3.41	5.40	25.75
% College	867	20.82	5.31	10.78	49.43
% Medicaid	867	14.90	4.64	5.30	31.78
% Medicare	867	15.06	2.60	7.19	23.88
% Private Insurance	867	68.37	6.48	48.76	85.11

Demographic variables are from the Current Population Survey. Data on CON laws are from the American Health Planning Association and the Mercatus Center. Data on substance use treatment facilities are from the National Survey of Substance Abuse Treatment Services

## Methods

We estimate fixed-effects regressions of the following form:

$${Y}_{st}={\beta}_0+{\beta}_1\ast {CON}_{st-1}+{\beta}_2\ast {\tau}_t+{\beta}_3\ast {S}_s+{\beta}_4\ast {X}_{st-1}+{\epsilon}_{st}$$

The dependent variables *Y*_*st*_ in various regressions include the natural log of facilities per 100,000 residents, natural log of beds per 100,000 residents, natural log of clients per 1000 residents, percent of facilities accepting Medicaid, percent of facilities accepting Medicare, and percent of facilities accepting private insurance. Taking natural log of facilities, beds, and clients corrects for the right-skewness (see Additional file 1: Appendix Figs. 1, 2 and 3 showing kernel density graphs of these variables before they are logged). *CON*_*st* − 1_, the key independent variable, indicates whether a CON law was in effect for SUD treatment facilities in the previous year. *τ*_*t*_ represents year fixed effects, *S*_*s*_ represents state fixed effects, and *X*_*st* − 1_ represents a vector of state-level control variables (PDMP law, insurance mandate law, state population, median age, mean income, percent white, percent Black, percent Asian, percent Hispanic, percent male, percent in poverty, percent with a college degree, percent with Medicaid, percent with Medicare, percent with private health insurance). State fixed-effects are included naturally by using the fixed-effects estimator, meaning that the coefficient for CON estimates the effect of a given state adding a substance use CON law.

## Results

Table 2 shows the results of the regressions described above. CON laws have no statistically significant effect on the number of facilities, beds, or clients and no significant effect on the acceptance of Medicare. The effect on Medicaid acceptance is not statistically significant at convetional levels. However, CON reduces the acceptance of private insurance by a statistically significant 6.0%.

**Table 2:** Predictors of substance abuse treatment capacity and payments

	(1)	(2)	(3)	(4)	(5)	(6)
	lnFacilities Per100k	lnBeds Per100k	lnClients Per1k	Accept Private	Accept Medicare	Accept Medicaid
CON	0.0204	0.0886^*^	0.0488	−0.0603^***^	−0.00862	− 0.0304^*^
	(0.0301)	(0.0535)	(0.0483)	(0.0115)	(0.0124)	(0.0174)
PDMP	0.0254	0.152^***^	0.129^***^	0.00859	−0.0312^***^	0.00651
	(0.0184)	(0.0394)	(0.0328)	(0.00703)	(0.00754)	(0.0106)
Insurance	0.0417^**^	−0.0194	0.0354	0.0145^**^	0.0217^***^	0.000663
Mandate	(0.0171)	(0.0340)	(0.0287)	(0.00653)	(0.00700)	(0.00987)
Pop (mil)	−0.0515^***^	−0.0468^***^	− 0.0903^***^	0.0116^***^	− 0.00910^**^	− 0.00215
	(0.00945)	(0.0180)	(0.0157)	(0.00361)	(0.00388)	(0.00546)
Median Age	0.00436	0.00638	0.0733^***^	0.0313^***^	0.00537	−0.0191^***^
	(0.00900)	(0.0172)	(0.0150)	(0.00344)	(0.00373)	(0.00518)
Income	0.00282	−0.00375	0.0663^***^	0.0308^***^	0.00733^**^	−0.0195^***^
	(0.00904)	(0.0172)	(0.0150)	(0.00346)	(0.00371)	(0.00523)
% White	−0.00911^***^	−0.00339	− 0.000314	0.00155	0.00101	−0.00471^***^
	(0.00253)	(0.00469)	(0.00419)	(0.000970)	(0.00104)	(0.00147)
% Black	0.00286	−0.00472	−0.000229	0.00192	0.00274^**^	−0.00452^**^
	(0.00325)	(0.00600)	(0.00520)	(0.00124)	(0.00133)	(0.00188)
% Hispanic	0.0353^***^	0.0321^***^	0.0456^***^	0.00317	0.00164	−0.0122^***^
	(0.00561)	(0.0103)	(0.00920)	(0.00215)	(0.00230)	(0.00324)
% Asian	0.00503	−0.00791	−0.00715	0.00283^**^	0.00326^**^	0.00617^***^
	(0.00327)	(0.00598)	(0.00530)	(0.00125)	(0.00134)	(0.00189)
% Male	−0.0132^***^	−0.00303	− 0.00572	0.00383^***^	0.000747	−0.000545
	(0.00382)	(0.00669)	(0.00603)	(0.00146)	(0.00157)	(0.00221)
%BelowPov	−0.0183^**^	0.000955	− 0.0100	− 0.00241	0.00454	0.000614
	(0.00905)	(0.0163)	(0.0145)	(0.00346)	(0.00371)	(0.00523)
% College	−0.00856^**^	− 0.0120^*^	− 0.0227^***^	− 0.000005	− 0.000953	0.00261
	(0.00334)	(0.00610)	(0.00538)	(0.00128)	(0.00137)	(0.00193)
% Medicaid	0.000843	0.00905	0.00261	−0.000362	− 0.000256	− 0.000578
	(0.00379)	(0.00703)	(0.00625)	(0.00145)	(0.00156)	(0.00219)
% Medicare	−0.00409^*^	0.00141	−0.00317	0.00364^***^	0.00328^***^	0.00635^***^
	(0.00236)	(0.00455)	(0.00396)	(0.000901)	(0.000967)	(0.00136)
% Private	0.00313	0.000711	0.0200^***^	−0.000159	− 0.000515	0.00176
	(0.00446)	(0.00824)	(0.00724)	(0.00171)	(0.00183)	(0.00258)
Year FE	Y	Y	Y	Y	Y	Y
State FE	Y	Y	Y	Y	Y	Y
Observations	918	763	813	918	918	918
R^2^	0.246	0.146	0.328	0.389	0.133	0.484

Standard errors in parentheses. Mean income is measured in thousands, population in millions

*FE* stands for Fixed Effects, *Y* stands for Yes

^***^p < 0.01

^**^p < 0.05

^*^p < 0.1

Table 3 repeats the regressions from Table 2, but includes a state-specific linear time trend in each one. Results remain similar; we still find that CON is associated with a statistically significant reduction in the acceptance of private insurance, now slightly larger at 6.25%. We still find no other effects of CON at conventional levels of statistical significance.

**Table 3:** Predictors of substance abuse treatment capacity and payments with state-specific time trends

	(1)	(2)	(3)	(4)	(5)	(6)
	lnFacilities Per100k	lnBeds Per100k	lnClients Per1k	Accept Private	Accept Medicare	Accept Medicaid
CON	−0.0544^*^	0.00897	0.0147	−0.0625^***^	− 0.0183	− 0.0312^*^
	(0.0298)	(0.0721)	(0.0592)	(0.0140)	(0.0136)	(0.0173)
PDMP	0.0330^**^	0.0683	0.130^***^	0.0156^**^	−0.00852	0.0167^*^
	(0.0161)	(0.0472)	(0.0356)	(0.00753)	(0.00732)	(0.00933)
Insurance	0.0219	0.0166	−0.0198	−0.00394	− 0.0197^***^	− 0.0242^**^
Mandate	(0.0166)	(0.0439)	(0.0351)	(0.00779)	(0.00757)	(0.00964)
Pop (mil)	−0.116^***^	− 0.0774	− 0.155^*^	−0.0228	− 0.0187	−0.00105
	(0.0415)	(0.101)	(0.0819)	(0.0194)	(0.0189)	(0.0241)
Median Age	0.00827	0.0392	0.0207	0.0236^***^	0.0177^***^	0.0136^*^
	(0.0135)	(0.0328)	(0.0270)	(0.00632)	(0.00615)	(0.00783)
Income	0.000454	−0.00653	−0.00102	0.00159	0.00254^**^	0.000619
	(0.00269)	(0.00681)	(0.00562)	(0.00126)	(0.00122)	(0.00156)
% White	−0.000870	− 0.00638	− 0.00534	0.00202^*^	0.00369^***^	− 0.00209
	(0.00245)	(0.00629)	(0.00492)	(0.00115)	(0.00112)	(0.00142)
% Black	0.0106^*^	−0.00773	− 0.00731	0.00520^*^	0.0114^***^	0.00571
	(0.00625)	(0.0150)	(0.0123)	(0.00293)	(0.00284)	(0.00363)
% Hispanic	0.00168	0.00347	−0.00644	−0.00179	0.000753	0.000133
	(0.00268)	(0.00666)	(0.00535)	(0.00126)	(0.00122)	(0.00156)
% Asian	−0.00670^**^	0.000864	−0.00476	0.000285	0.000008	−0.000565
	(0.00297)	(0.00749)	(0.00601)	(0.00139)	(0.00135)	(0.00172)
% Male	−0.00436	− 0.00591	0.00623	−0.000159	0.00475	0.00644
	(0.00723)	(0.0177)	(0.0144)	(0.00339)	(0.00329)	(0.00420)
%BelowPov	−0.00167	− 0.0126^**^	− 0.0142^***^	0.000457	− 0.00201^*^	0.00156
	(0.00241)	(0.00618)	(0.00487)	(0.00113)	(0.00110)	(0.00140)
% College	0.00573^**^	0.0152^**^	0.00254	0.000865	0.000405	0.00127
	(0.00287)	(0.00725)	(0.00585)	(0.00134)	(0.00131)	(0.00166)
% Medicaid	0.00143	0.00704	0.000628	0.00122	0.00117	0.00287^**^
	(0.00200)	(0.00530)	(0.00422)	(0.000935)	(0.000909)	(0.00116)
% Medicare	0.00198	0.00289	0.0196^***^	−0.00185	−0.00240	−0.00187
	(0.00330)	(0.00837)	(0.00666)	(0.00154)	(0.00150)	(0.00191)
% Private	0.00225	0.000895	0.00137	0.000009	−0.000172	− 0.000001
	(0.00186)	(0.00485)	(0.00387)	(0.000870)	(0.000845)	(0.00108)
Year FE	Y	Y	Y	Y	Y	Y
State FE	Y	Y	Y	Y	Y	Y
State Time Trend	Y	Y	Y	Y	Y	Y
Observations	918	763	813	918	918	918
R^2^	0.672	0.309	0.550	0.601	0.535	0.774

Standard errors in parentheses. Mean income is measured in thousands, population in millions

*FE* stands for Fixed Effects, *Y* stands for Yes

^***^p < 0.01

^**^p < 0.05

^*^p < 0.1

## Conclusion

States adding substance use CON laws are associated with a lower likelihood of facilities accepting private insurance, with no statistically significant effect on the number of facilities, beds, or clients per capita and no significant effect on acceptance of Medicare or Medicaid. These results are somewhat puzzling, as we expected that the mechanism by which CON laws lead to fewer forms of payment being accepted is by reducing the number of facilities in the market and so reducing competition. But we found no significant effect on the number of facilities. When controlling for state-specific time trends the estimated effect for facilities did turn negative and close to significant, while the coefficients on beds and clients were positive and the coefficients for all forms of payment were negative. These coefficients are consistent with CON leading to fewer but larger facilities, with each facility being more selective about which forms of payment the accept. But with our data and empirical strategy, only the reduction in acceptance of private insurance is statistically significant at conventional levels.

One limitation of our study is that endogeneity is pervasive in this setting: states might pass or repeal CON laws based on their expectations of the need for treatment facilities, and the demand for care varies with substance use levels, which we do not control for directly and which themselves may depend on the availability and effectiveness of treatment facilities.

A further limitation, shared by most work on substance use, is that we rely on surveys for our key variables. Correspondence with the data-set creators concerning potential mistaken responses noted that “N-SSATS is a voluntary survey that substance use facilities complete to the best of their ability based on their understanding of the questions.. .. we have noted this possible discrepancy and will consider implementing procedures to identify and impute these variables in future surveys.” Likewise, we noted discrepancies between two versions of the AHPA data on substance use CON laws; AHPA advised us that the “matrix” version we used should be trusted over the map version, which has since been removed from their website. Future work should consider the use of administrative data where possible.

While these limitations mean that our estimates lack precision, it remains the case that the evidence for CON requirements for SUD treatment facilities is either nonexistent or negative. The stated intention of CON laws is to promote access to care [8], but the only previous study on them [8] found that they reduce the number of SUD facilities, and we find that their only significant effect is to reduce the forms of payment accepted by facilities. Given that taxpayers finance a large share of the costs associated with SUDs through the funding of public insurance programs, free treatment paid through government grants and contracts, and cost shifting, policy makers may wish to reconsider whether substance use CON laws are promoting their intended goals.

## Supplementary Information

**Additional file 1: Appendix Table 1. Appendix Figure 1.** Facilities Per 100,000 Residents in a State. **Appendix Figure 2.** Beds Per 100,000 Residents in a State. **Appendix Figure 3.** Clients Per 1000 Residents in a State.

## Data Availability

Datasets used in this study are available at https://osf.io/eswxt/

## Abbreviations

AHPA: American Health Planning Association
CON: Certificate of Need
N-SSATS: National Survey of Substance Abuse Treatment Services
PDMP: Prescription Drug Monitoring Programs
SAMHSA: Substance Abuse and Mental Health Services Administration
SUD: Substance Use Disorder
