# Supplementary material for: Certificate-of-need laws and substance use treatment
Source: Subst Abuse Treat Prev Policy. 2022 May 18;17:38. doi: 10.1186/s13011-022-00469-z (PMC9118675; doi:10.1186/s13011-022-00469-z)
Supplement: Supplementary file 1 — Additional file 1: Appendix Table 1. Appendix Figure 1. Facilities Per 100,000 Residents in a State. Appendix Figure 2. Beds Per 100,000 Residents in a State. Appendix Figure 3. Clients Per 1000 Residents in a State. [file 13011_2022_469_MOESM1_ESM.docx]

**Supplementary Information**

**Additional file 1**

**Appendix 1**

When data sources conflicted about which states had substance use CON in which years, we turned to state statutes and regulations. The report by the Institute for Justice cites precise statutes and regulations to back its claims, and the Mercatus Center shared similar precise citations when asked about discrepancies with AHPA. The non-AHPA sources show more states with substance use CON laws (e.g., 23 states in the 2020 Mercatus data vs. 17 in the 2016 AHPA data). It appears that these discrepancies are driven by errors in the AHPA data, as opposed to states enacting CON laws between 2016 and 2020. Checking state statutes and regulations revealed that each state listed by AHPA as non-CON and by Mercatus as CON actually had a substance use CON law since at least 2002; we have updated our data to reflect this, and we cite the statues for these states below.

**Appendix Table 1**

| State | Legal Citation for Substance Use CON |
| --- | --- |
| Missouri | (Regulation 19 CSR 60–50.440) |
| New York | (Mental Hygiene Law Chapter 27 Title E section 32.09) |
| Oklahoma | (Title 63, Chapter 1, Article 8, section 1–880.5) |
| Oregon | (Regulations 333–550–000 and 333–550–0010) |
| Virginia | (section 32.1–102.11) |
| Washington | (section 71.12.455) |

**Appendix 2**

**Appendix Figure 1: Facilities Per 100,000 Residents in a State**

**Appendix Figure 2: Beds Per 100,000 Residents in a State**

**Appendix Figure 3: Clients Per 1,000 Residents in a State**
